# Host genotype, soil composition, and geo-climatic factors shape the fonio seed microbiome

**DOI:** 10.1186/s40168-023-01725-5

**Published:** 2024-01-17

**Authors:** Naheed Tabassum, Hanin Ibrahim Ahmed, Sabiha Parween, Arsheed H. Sheikh, Maged M. Saad, Simon G. Krattinger, Heribert Hirt

**Affiliations:** 1 Plant Science Program, Biological and Environmental Science and Engineering Division (BESE), King Abdullah University of Science and Technology (KAUST), 23955-6900 Thuwal, Saudi Arabia; 2 Center for Desert Agriculture, Biological and Environmental Science and Engineering Division (BESE), King Abdullah University of Science and Technology (KAUST), 23955-6900 Thuwal, Saudi Arabia

**Keywords:** Seed microbiome, Core microbiome, Microbiome engineering, Plant stress adaptation

## Abstract

**Background:**

Fonio (*Digitaria exilis*), an orphan millet crop, is the oldest indigenous crop in West Africa. Although the yield is low due to pre-domestication characteristics, the quick maturation time, drought tolerance, and the ability to thrive on poor soils make fonio a climate-smart crop. Being holobionts, plants evolve in close interaction with microbial partners, which is crucial for plant phenology and fitness. As seeds are the bottleneck of vertically transmitting plant microbiota, we proposed to unravel the seed microbiome of the under-domesticated and resilient crop fonio. Our study investigated the bacterial seed endophyte diversity across 126 sequenced fonio accessions from distinct locations in West Africa. We conducted a correlation study of the structures and functions of the seed-associated microbiomes with the native geo-climate and soil structure data. We also performed Genome-wide association studies (GWAS) to identify genetic loci associated with seed endophyte diversity.

**Result:**

We report that fonio millet has diverse heritable seed endophytic taxa. We analyzed the seed microbiomes of 126 fonio accessions and showed that despite the diversity of microbiomes from distinct geographical locations, all fonio genetic groups share a core microbiome. In addition, we observed that native soil composition, geo-climatic factors, and host genotype correlate with the seed microbiomes. GWAS analysis of genetic loci associated with endophyte seed bacterial diversity identified fonio SNPs associated with genes functioning in embryo development and stress/defense response.

**Conclusion:**

Analysis of the seed endophyte of the climate-smart crop fonio indicated that despite possessing a heritable core microbiome, native conditions may shape the overall fonio seed microbiomes in different populations. These distinct microbiomes could play important roles in the adaptation of fonio to different environmental conditions. Our study identified the seed microbiome as a potential target for enhancing crop resilience to climate stress in a sustainable way.

Video Abstract

**Supplementary Information:**

The online version contains supplementary material available at 10.1186/s40168-023-01725-5.

## Background

 Fonio (*Digitaria exilis*) is one of the oldest, indigenous, semi-domesticated, and fastest-maturing cereals in West Africa [23, 27]. Fonio can grow in nutrient-poor soil and is very drought tolerant, which makes it suitable for dryland agriculture [7]. Nutritionally, fonio is comparable to other cereals [60]. It could complement the staple crops amid climate change and desertification challenges. Due to some wild characteristics, including seed shattering and lodging, fonio yields are much lower compared to the major cereal crops rice, maize, and wheat [8]. Fonio is a tetraploid species (2*n* = 4 ×  = 36) with a high inbreeding rate. Population genomic analyses revealed that the genetic diversity in fonio is relatively low [6]. Recently, a fonio high-quality, chromosome-scale reference assembly and whole-genome sequencing data of a fonio diversity panel were established and analyzed [5]. The diversity panel included 183 wild and semi-domesticated accessions covering a wide geographic and climatic range. The fonio population structure was shaped by climatic, geographic, and ethnolinguistic factors, and the 183 fonio accessions were divided into six distinct genetic clusters/groups and one admixed group.

Besides harnessing genetic information and modification, breeders can improve plants by exploring and maneuvering their association with microbes [31]. For instance, plants grown in arid conditions associate and interact with bacteria that help plants combat abiotic challenges [17]. Such plant–microbe interaction symbiotically attains a high degree of adaptation to the extreme environment. In addition, the microbiome plays a crucial role in plant growth and development, nutrient uptake, and protection against biotic stress. Arguably, plants are holobionts meaning that a community of microbes coevolves with their host contributing to host phenology and fitness [38]. Therefore, microbes perform crucial functions throughout plant life history [49].

The selection and assembly of the plant microbiome is a complex, multistep, successional process. Members of horizontally (from the surrounding niche) or vertically (from the mother plant) acquired microbiota can contribute to the ultimate composition of plant microbiota [49, 52]. The embryo surface, storage tissue, and seed coat represent distinct seed microhabitats. Seeds harbor fewer microbial taxa than other plant tissues, and the number of seed microbes varies among plant species [1]. The vertical transmission of potentially beneficial microbial communities to fortify the progeny of a plant species opens a new technological application in crop breeding [9]. Seed microbe engineering could be used for targeted metabolites or antimicrobial compound production to improve plant biomass and yield under biotic or abiotic stresses [50].

A core microbiota is associated with a host in broad-ranging environmental conditions [55]. Compared to adult plants (with rhizosphere), the core microbes are likely to be more reproducibly found in seeds and seedlings [54]. For example, the core endophytic microbiota of maize is conserved in seeds across boundaries of evolution, ethnography, and ecology [29]. Also, maize seeds constitute a significant source of inoculum for juvenile root endophytes [28]. It has been recently proposed that one should describe the potential functional relevance of core microbiota rather than their taxonomy [59]. The dynamics of vertically transmitted core seed microbiota were also shown in six rice varieties [59], but the study of their functions is yet unexplored.

Here we investigated the seed-associated microbiome of an underutilized, resilient orphan crop. We report that 126 fonio accessions have diverse seed endophytic microbiomes. The six and admixed fonio genetic groups were found to share a heritable core seed microbiome. Moreover, we observed that native soil composition, geo-climatic factors, and host genotype correlate with the seed microbiomes of different fonio accessions. Finally, using a genome-wide association study (GWAS) for endophyte seed bacterial diversity, we found that distinct fonio genetic loci are associated with genes with roles in embryo development and stress/defense response. Our study provides a proof of concept to understand the composition and diversity of the seed-associated microbiomes of a cereal from distinct geographical locations. Furthermore, our study highlights the potential of the seed microbiome as a target for microbiome engineering in future crop breeding programs.

## Materials and methods

### Germplasm

**Fig. 1:**
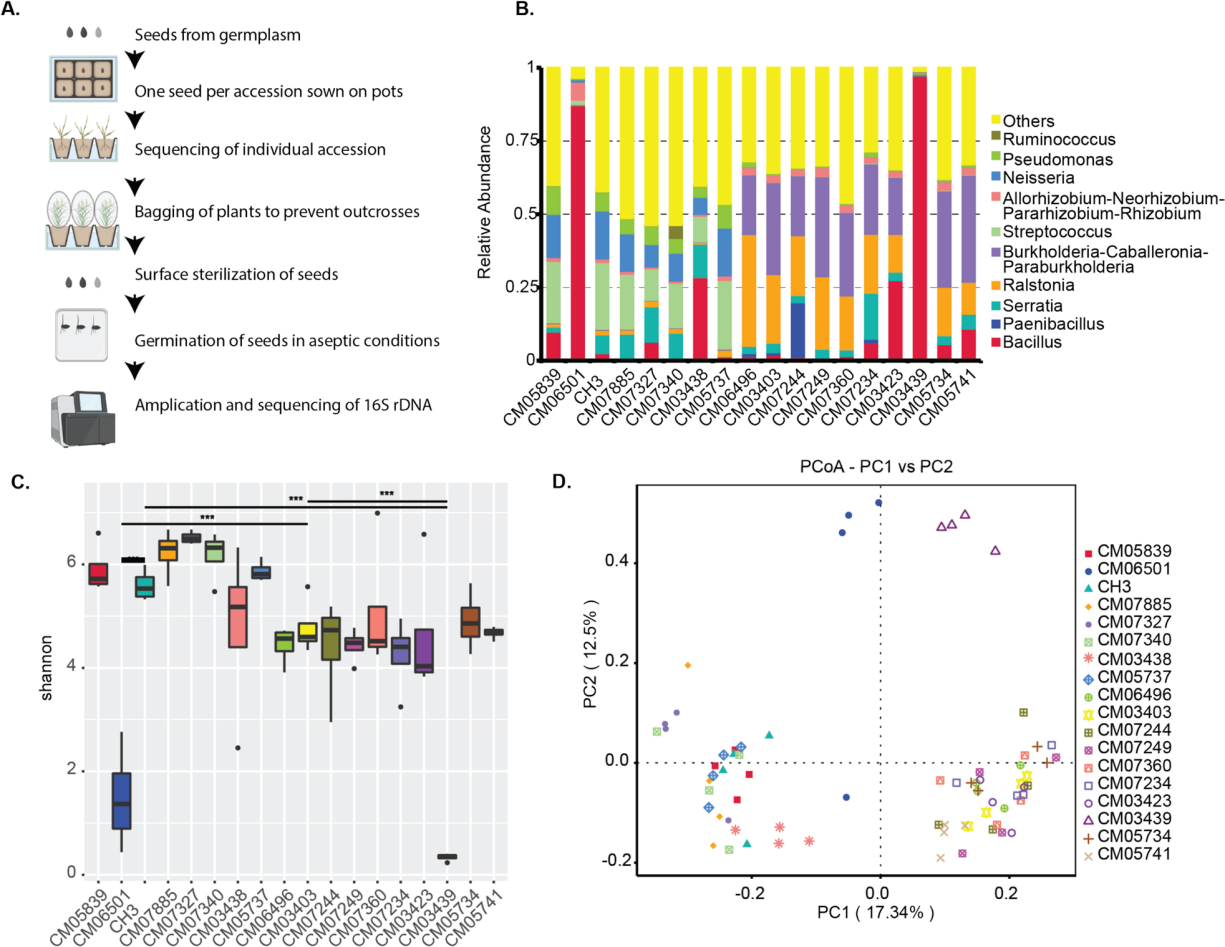
Diversity of seed-associated microbiomes in 18 fonio accessions. **A** Scheme showing sample collection and processing in the study. We germinated a single seed per accession and used the progeny seed for sterilization, germination, 16S rDNA sequencing and analysis. (Illustration created by Biorender.com) Histograms of the relative abundances of the top 10 bacterial communities at genus level (**B**) and Boxplot of Shannon index showing diversity in the 18 fonio accessions (**C**). Group 1 (CM06501, CM05839, CM06496), group 2 (CM07885, CH3, CM03403), group3 (CM07249, CM07244, CM07327) and group 4 (CM07340, CM07234, CM07360), and group 5 (CM03438, CM03423, CM03439) and group 6 (CM05737, CM05741, CM05734). Kruskal–Wallis test, * indicates *p* < 0.05, ** indicates *p* < 0.01 and, and *** indicates *p* < 0.001. The box boundaries indicate the first and third quartile. Lines extending from the boxes (whiskers) indicate variability outside the lower and upper quartiles The lines in the middle of the boxes represent the median values of Shannon index for accession. Outliers are plotted as individual points. **D** Principal component analysis (PCoA) shows the grouping patterns of the accession based on unweighted UniFrac distance

Seeds from a diversity panel described by Abrouk et al. [5] were used. We germinated a single seed per accession simultaneously in the same soil in the greenhouse (Fig. 1A). The resulting seeds from the propagated single plants of 126 accessions (118 accessions with 3 replicates and 8 accessions with 2 replicates) were then used in the pilot and main studies. For vertical transmission, we used the seeds from the original and the propagated plants from three accessions.

### Extraction of DNA from seedlings and amplification of bacterial 16S rDNA

We surface sterilized the seeds using 70% ethanol + 0.05% sodium dodecyl sulfate for 10 min on a shaker, washed 2 times in 96% ethanol, and let to dry. We germinated surface sterilized seeds on 0.5 MS media (MS basal salts, Sigma). The seedlings were harvested with liquid nitrogen in sterile condition using sterilized equipment including tubes with closed lid and pulverized. We performed crude extraction of genomic DNA by adding an extraction buffer (100 mM Tris pH = 8, 50 mM EDTA, 500 mM NaCl, 1.5% SDS). We mixed the lysate with 1:1 vol of isopropanol and centrifuged at 14,000 rpm for 10 min. We washed the DNA pellets with 70% ethanol, dried and dissolved them in 1X TE buffer (10 mM Tris, 1 mM EDTA, pH 8.0). We checked the purity of the DNA using NanoDrop (Thermo) and qubit (Thermo).

### Amplicon generation and illumina sequencing

We amplified 16S rDNA using V5-V7 (799F/1193R) primers with the barcode (799F, 5′-AACMGGATTAGATACCCKG-3′, 1193R, 5′-ACGTCATCCCCACCTTCC-3′). We performed the PCR reaction with Phusion® High-Fidelity PCR Master Mix (NEB) (including a negative control). We checked the PCR products on 2% agarose gel, and 400–450 bp amplicons were mixed in an equal-density ratio and then purified the PCR product with a Qiagen Gel Extraction Kit (Qiagen). We generated the sequencing libraries using NEB Next® Ultra DNA Library Prep Kit for Illumina and added the index codes, following the manufacturer’s recommendations. We assessed the library quality on the Qubit@ 2.0 Fluorometer (Thermo Scientific) and Agilent Bioanalyzer 2100 system. At last, we sequenced the library on an Illumina platform and generated 250 bp paired-end reads.

### Bioinformatic analysis

#### Paire-end reads assembly and quality control

Paired-end reads were assigned to samples based on their unique barcodes and truncated by cutting off the barcode and primer sequences. We used FLASH (v1.2.11, http://ccb.jhu.edu/software/FLASH/) to merge the reads to get raw tags after removing the barcode and primer sequence. We used fastp software to obtain high-quality clean tags. We then blast clean tags to the database using Vsearch software. We removed the chimera to obtain final effective tags.

#### Cluster/denoise and species annotation of amplicon sequence variants (ASVs)

We used DADA2 or deblur module in QIIME2 software to denoise and obtain the final ASVs. Then, we used the Classify-sklearn moduler in QIIME2 software to compare ASVs with the database and to obtain the species annotation of each ASV. The absolute abundance of individual microbial taxa within a sample was determined by quantifying the number of reads or sequences associated with each ASV. Greater numbers of assigned sequences to an ASV corresponded to increased absolute abundance within the sample.

#### Alpha diversity

We apply alpha diversity in analyzing the complexity of species diversity with six indices, including observed species, chao1, Shannon, Simpson, ACE, and goods-coverage (http://www.mothur.org). All these indices in our samples were calculated with QIIME (Version 1.7.0) and displayed with R software (Version 2.15.3).

#### Beta diversity

We used beta diversity analysis to evaluate differences of samples in species complexity. We calculated beta diversity on both weighted and unweighted UniFrac distances by QIIME software (Version 1.7.0). We performed PCoA analysis) to get principal coordinates and visualize complex multidimensional data using the WGCNA package, stat packages, and ggplot2 package in R software (Version 2.15.3). We obtained a distance matrix of weighted or unweighted UniFrac among samples obtained before transforming to a new set of orthogonal axes.

#### Functional prediction of core ASVs

We predicted the core microbiome’s function using PICRUSt2 (Phylogenetic Investigation of Communities by Reconstruction of Unobserved Stats 2) software using the default “max parsimony” method for hidden-state prediction and a Nearest Sequenced Taxon Index (NSTI) value of 2.0. The Kyoto Encyclopedia of Genes and Genomes (KEGG) orthologs (KO) abundances predictions were obtained, and its description were obtained from the KofamKOALA-KEGG Orthology Search database.

#### Phylogeny of core ASVs

The phylogenetic analysis of 191 core ASVs was done using the PHYLIP program at default mode. The tree was visualized using Tree Viewer and each of the ASVs’ absolute abundance values was highlighted.

#### Genome-wide association study of fonio microbiome

A genome-wide SNP map of fonio used in this study was generated previously based on the whole-genome sequencing of the fonio diversity panel [5]. To find the significant genetic variants in the host that are associated with the microbiome, we considered the first axis of the UniFrac weighted PCoA of one replicate as phenotypes (i.e., β-diversity) and SNP data from 99 fonio accessions as genotypes. The 99 accessions were selected based on replicates clustering together on the PCoA analysis. When all three replicates from a particular accession did not exhibit coherent clustering patterns, indicating potential inconsistencies, we excluded that specific sample from the GWAS analysis to ensure the reliability and coherence of our association analysis. We then filtered SNPs with 5% Minor allele frequency (MAF), 10% missing rate using VCFtools (v0.1.17) [15] and Linkage disequilibrium (LD) with *R*^2^ ≥ 0.3 (using PLINK software v1.90 [45] resulting in 137,476 SNPs. We tested the association of SNPs with microbiome variability by the general linear model (GLM) using three principal components as covariates to control for population structure. We identified the significant associations above the threshold of Bonferroni *p*-value = 1e − 05. We then considered a genomic window of 50 kb upstream and 50 kb downstream for each position as a candidate region. To test the relationship between microbiome variability and environmental factor, Pearson’s correlation test was performed using MVapp (https://mvapp.kaust.edu.sa/).

## Results

### Fonio accessions have diverse seed endophytic microbiomes

The fonio genetic population structure was previously defined by six distinct and one admixed genetic group [5]. Fonio accessions were grown for one generation in the greenhouse and in the same soil to minimize any experimental bias (Fig. 1A). For a pilot study, we randomly selected three accessions from the six genetic and one admixed group to understand the composition of the seed endophytic microbiome. These accessions belong to group 1 (CM06501, CM05839, CM06496), group 2 (CM07885, CH3, CM03403), group3 (CM07249, CM07244, CM07327), group 4 (CM07340, CM07234, CM07360), group 5 (CM03438, CM03423, CM03439), and group 6 (CM05737, CM05741, CM05734). We aseptically germinated surface sterilized seeds of the 18 accessions and amplified 16S rDNA from gDNA (4 replicates). Using the ASV (Amplicon Sequence Variant) approach, we obtained a total of 11.4 million reads that could be assembled into 13,970 ASVs of bacterial taxa (Table S1). The rarefaction curves showed that the sequence library size was sufficient to cover the microbial diversity in each sample (Fig. S1A). The dominant genera were *Bacillus*, *Paenibacillus*, *Serratia*, *Ralstonia*, *Streptococcus*, *Burkholderia*, *Neisseria*, and *Pseudomonas* (Fig. 1B, Table S2). Next, we calculated the alpha diversity of the observed species using the Shannon index. While two accessions CM03439 and CM06501 have lower alpha diversity with the predominance of *Bacillus*, others have high alpha diversity (Fig. 1C, Fig. S1B Table S3). To further gain insights into the microbiota structures, we used PCoA plots based on unweighted UniFrac distance to visualize the separation of the microbiota structures in these 18 accessions, each with four replicates (Fig. 1D). We observed that CM03439 and CM06501 with lower diversity clearly differentiated, while accessions with moderate and high diversity are partially differentiated. Overall, this pilot study showed that the seed bacterial endophytic microbiomes of different fonio accessions are potentially composed of unique and shared taxa.

### Diverse genetic fonio groups share a core microbiome

**Fig. 2:**
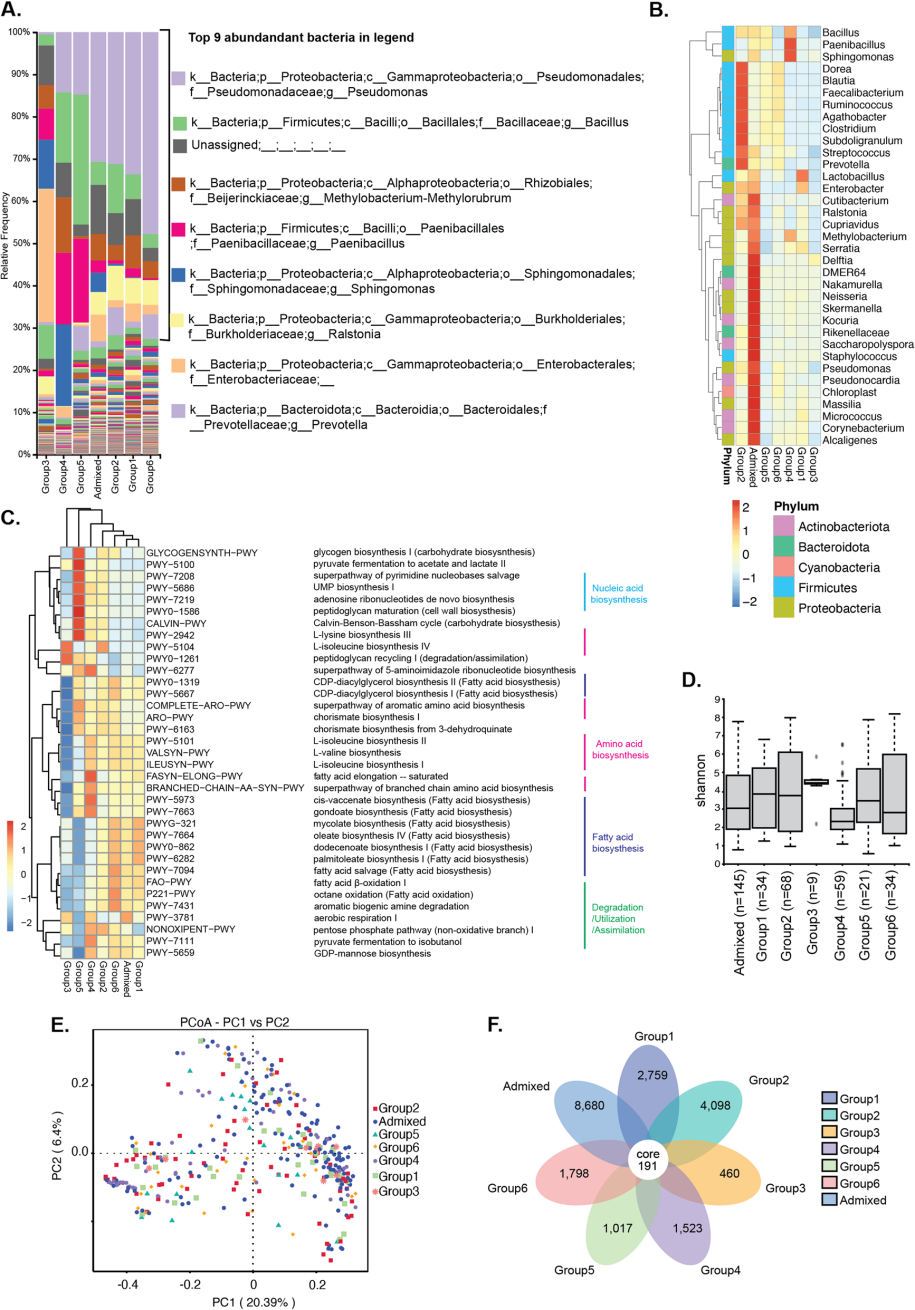
Core and diverse ASVs across the fonio genetic population. **A** Histograms of the relative abundances of the top bacterial communities at genus level in the fonio genetic group (admixed, group 1, group 2, group 3, group 4, group 5, and group 6. Heat map showing over/underrepresented bacteria at genus level (**B**) and functional predictions across fonio accession groups (**C**). **D** The box plots show the Shannon diversity index across the genetic fonio groups where “n” is the number of samples. The box boundaries indicate the first and third quartile. Lines extending from the boxes (whiskers) indicate variability outside the lower and upper quartiles. The lines in the middle of the boxes represent the median values of Shannon index for each group. Outliers are plotted as individual points. **E** PCoA of 126 fonio accessions (color and shape code is based on the corresponding groups) based on unweighted UniFrac distance. **F** The flower diagram shows unique and core taxa with the admixed group

The fonio genetic population structure correlated with geo-climatic factors and was defined by six distinct and one admixed genetic group [5]. We used 126 fonio accessions representing these six genetic and one admixed groups for an in-depth analysis of the structure and function of the seed-associated microbiomes (Fig. S2A, Table S4). Using the ASV approach, 73.8 million reads were assembled into 30,356 ASVs (unfiltered) of bacterial taxa (Table S5). The rarefaction curves (Fig. S2B) showed that the sequence library size was sufficient to cover the microbial diversity in each group. Overall, the dominant phyla across the fonio genetic groups were Proteobacteria and Firmicutes (Fig. S2C), whereas the dominant genera were *Pseudomonas* and *Bacillus* (Fig. 2A). Interestingly, the genera *Lactobacillus* and *Enterobacter* are overrepresented in fonio group 1, whereas group 2 exhibits a significant overrepresentation of the Firmicutes phylum (Fig. 2B). While fonio group 4 stands out for its specific overrepresentation of the genera *Bacillus*, *Paenibacillus*, and *Sphingomonas* the admixed group showed a pronounced overrepresentation of the phyla Proteobacteria and Actinobacteriota (Fig. 2B).

To gain insights into the metabolic potential of the microbiome, we conducted a Picrust2 analysis, from which we inferred the top 35 pathways (MetaCyc) based on 16S rRNA gene sequences (Fig. 2C). Among these pathways, we observed both anabolic processes, such as the biosynthesis of fatty acids, nucleic acids, and amino acids, as well as catabolic pathways involved in degradation/utilization. While the functional roles did not result in clear clusters among the groups in the PCA plot (Fig. S2D), we did identify instances of over/under representation of specific functions among different groups. These findings suggest that the functions performed by seed-associated endophytes could potentially contribute to plant growth and development through nutrient availability and help to adapt phenology and fitness to the respective environmental conditions as the prominence of these pathways varied among the fonio genetic groups.

To assess the species richness and diversity (alpha diversity), we analyzed the Shannon indexes among the fonio genetic groups revealing variation across the genetic groups (Fig. 2D). For example, group 6, with a lower sample size (*n* = 34), is more diverse than group 2 (*n* = 68) or the admixed group (*n* = 145) with a higher sample size. We used PCoA plots based on unweighted UniFrac distance to visualize the microbiota structures. We did not observe any differentiation of fonio accessions that belong to the same genetic group, indicating that the microbiome composition is complex and potentially shares the taxa (Fig. 2E). Therefore, we took a reductionist approach [20] to understand the microbiome composition of each fonio genetic group. To visualize the abundance of unique and shared microbial core taxa in the six genetic and admixed groups, we generated a floral diagram (Fig. 2F). Here, the sample size in each genetic group is proportionate with their unique taxa. For example, group 2, with the highest number of samples, had the most abundant unique taxa. Despite coming from distinct geographical locations, all genetically distinct fonio groups shared 191 core bacterial taxa. Also, the admixed group, which shares genetic diversity with other groups, retains all the core microbiota but contains > 8600 unique bacterial taxa. A side-by-side comparison of Fig. S2E and F shows that the number of unique microbial taxa in each fonio genetic group is lower in Fig. 2F. This indicates that the admixed fonio group shares some microbial taxa with each non-admixed fonio group, the core taxa remain the same. Among the core, the majority of phyla are Proteobacteria (31%), Firmicutes (25%), Actinobacteriota (21%), Bacteroidota (8%), and unassigned/partially assigned/others (15%) (Table S6). Although the absolute abundance of each core taxa varied among the fonio genetic groups, the most abundant genera are *Pseudomonas* sp. (ASV0) (Fig. S3, Table S7). We further predicted the function of the core taxa in metabolism, signaling, and genetic information processing using KEGG ontology (KO) (Fig. S2F). The most prevalent KO included the terms peptidase and inhibitors, secretion systems, two core component systems, and transcription factors. We hypothesize that these functional traits possess plant-beneficial properties that may help the endophytes to establish the symbiosis with fonio.

**Fig. 3:**
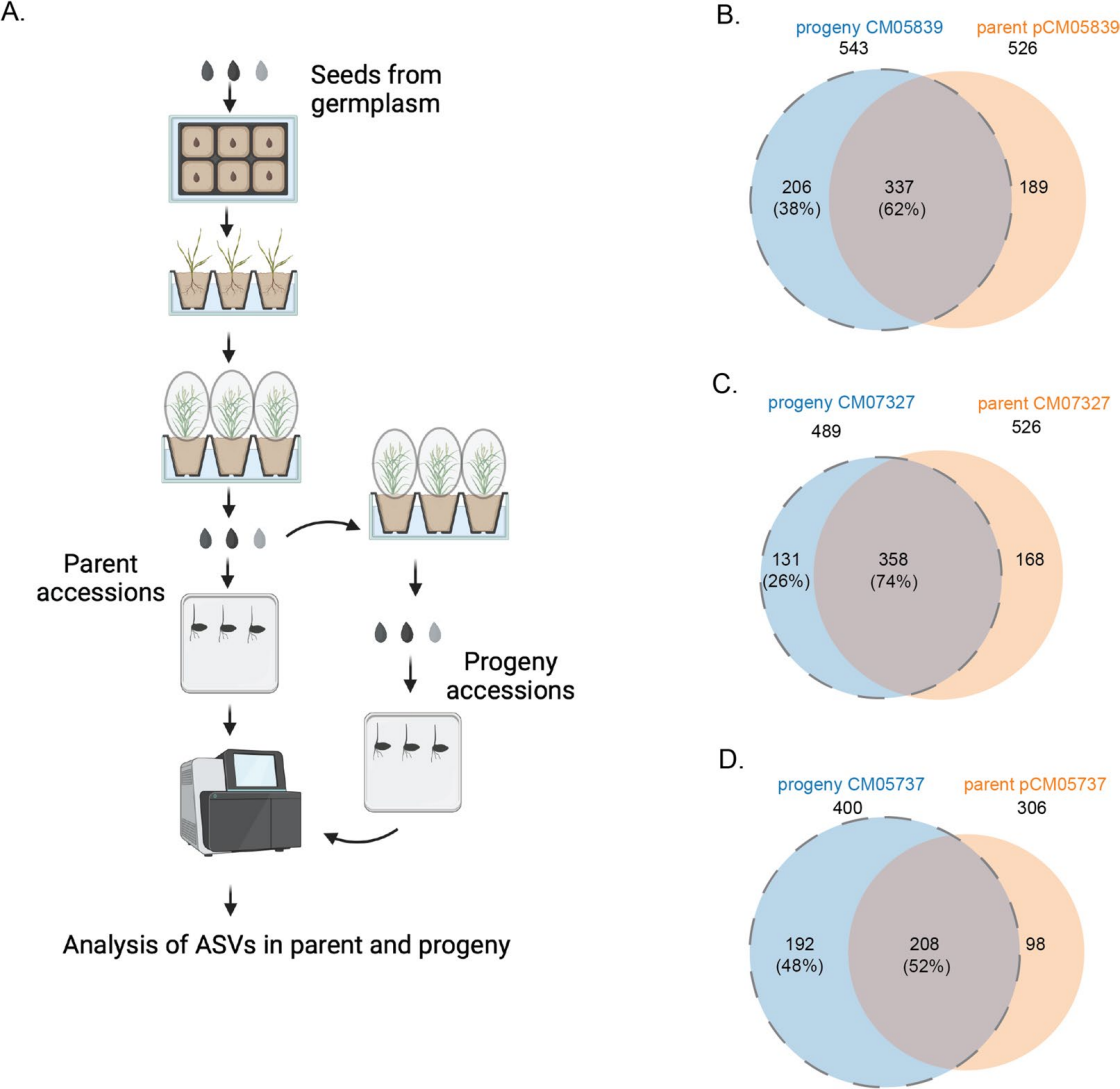
Heritable microbial taxa in 3 selected fonio accessions. **A** Scheme showing experimental set up to study vertical and horizontal transmission of ASVs parent and progeny (illustration created by Biorender.com). **B**–**D** Venn diagram of vertically transmitted ASVs in 3 fonio accessions: CM05839, CM07327, and CM05757. The accessions (parent accessions) were grown for one more generation to get the progeny seeds. These seeds were surface sterilized, germinated and 16S amplicon sequencing was performed

As seeds represent a bottleneck in inheriting microbiomes [2], we checked for the vertical transmission of the respective seed microbiome structures in three accessions (Fig. 3A). For this, we analyzed the presence of common ASVs in the parent and progeny seeds of CM05839 of group 1, CM07327 of group 3, and CM05757 of group 6. We observed that in accession CM05839, 62% (337/543 × 100) of progeny ASVs were acquired from parents, and 38% (206/543 × 100) of progeny ASVs were acquired from soil. (Fig. 3B). Likewise in accession CM07327 and CM05757, 74% (358/489 × 100) and 52% (208/400 × 100) of progeny ASVs were acquired from parent and 26% (131/489 × 100) and 48% (192/400 × 100) from soil, respectively (Fig. 3C and D, Table S8). Altogether, these data show that although a significant proportion of the seed microbiomes can be transferred vertically from seed to seed, the overall microbiome composition remains dynamic as a notable portion of ASVs can originate from the soil environment.

#### Soil composition, geo-climatic factors, and host genotype correlate with the fonio seed microbiome

**Fig. 4:**
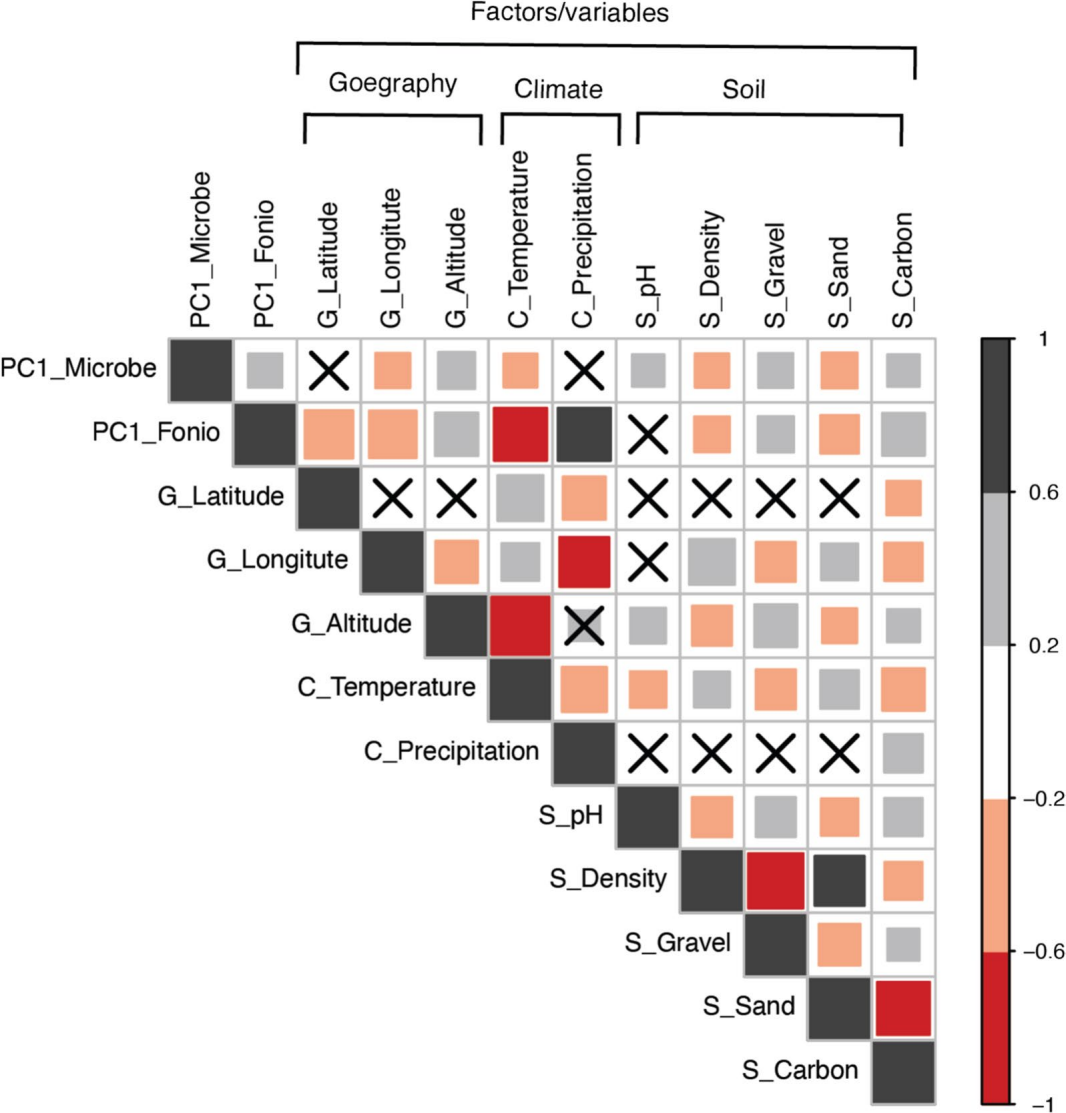
Correlation of seed microbiome diversity with environmental variables. The figure shows the Pearson correlation coefficients between selected traits. The color and size of the square reflect the strength of the correlation. The non-significant correlations, with a *p*-value above 0.01, are indicated with a cross in the individual cells. The correlation coefficients are calculated using raw data, with a total number of 126 fonio accessions. PC1_microbes are the first PC axis from the PCoA analysis based on weighted UniFrac distances, which represents the microbiome variability. PC1_Foniois the first PC axis from the PCA analysis based on SNP frequncies taken from Abrouk et al. that represents the fonio gentic diversity. G_Latitude, G_Longitude, and G_Latitude are the geographical factors (G). C_Temperature (annual mean temperature), and C_Precipitation (annual mean precipitation) are the climate conditions (C). S_pH: the topsoil pH in water. S_Desntiy: the topsoil bulk density. S_Gravel: the topsoil gravel content are soil variable (S)

As the fonio accessions are derived from distinct geo-climatic conditions but retain unique/hub microbiota (Fig. S4), we hypothesized that the environmental factors might influence the assembly, presence, and shape of the seed microbiomes. To test this hypothesis, we obtained surface soil data (pH, density, gravel, sand content) from their collection sites (Table S9). We observed a correlation (Pearson’s correlation; *p* < 0.01) between the microbial diversity (the first principal component of PCoA) and soil variables, including pH, gravel content, soil density, gravel, and carbon content (Fig. 4, Table S10). Because the fonio genetic diversity correlates with geographical (latitude, longitude, altitude) and climate factors (mean temperature and precipitation) [5], we asked whether the seed microbiomes show similar correlations. We found a significant correlation with longitude, altitude, and temperature but not with precipitation and latitude. However, the fonio genotypes also correlated with soil factors such as density, gravel, sand, and carbon content. In conclusion, we observed a positive correlation between the fonio genetic population structure and the diversity in their associated microbiomes. Hence, soil composition, geo-climatic factors, and the host genotype correlate with the seed microbiomes of different accessions (Fig. 6).

#### Genome-wide association study (GWAS) unveils fonio genetic loci associated with endophyte microbiome structure

**Fig. 5:**
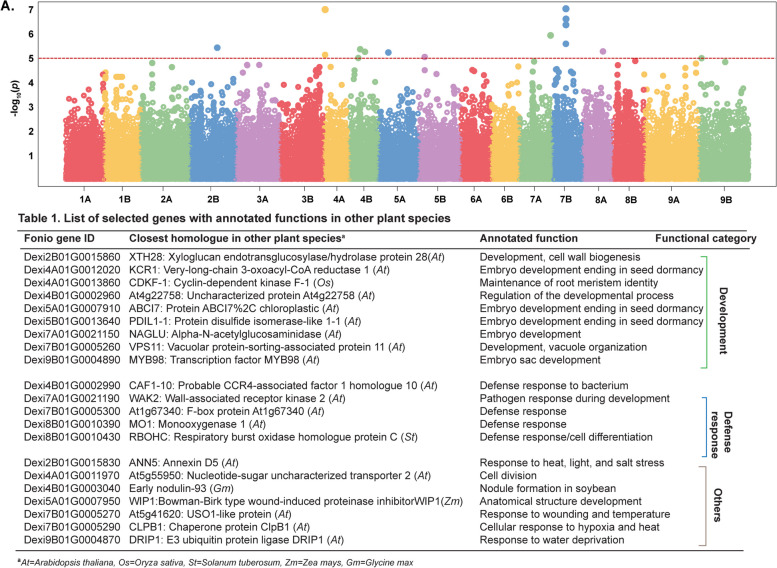
GWAS with the first axis of uniface weighted PCoA using a general linear model (GLM) and enrichment analysis. A The Manhattan plots display the association *p*-value for each SNPs in fonio. The red horizontal lines represent the *p* = 1e − 5 significance threshold. Table 1 List of the fonio gene ID, closest homolog in other species, annotated function with categories

The correlation between the genetic diversity of seed microbiomes with the genetic diversity of fonio (Fig. 4) is an ideal starting point to find genetic loci in fonio that are associated with the seed microbiome structure [18]. We performed GWAS to find associations between host genetic loci and seed microbiome diversity. Our GWAS with seed microbiome β-diversity revealed significant associations for 17 fonio SNPs on chromosomes 2B, 4A, 4B, 5A, 5B, 7A, 7B, 8B, and 9B (Fig. 5A, Fig. S5). Out of these, 16 SNPs were intergenic. As done previously, we considered 50 kb downstream and upstream of the SNP locus to obtain a list of candidate genes associated with the genetic locus [5], revealing 117 genes that might influence microbiome β-diversity. One prominent candidate interval on chromosome 7B contained ten genes (Fig. 5A, Table 1, Table S11), including a homolog of the *Arabidopsis* At*Vps11* (vacuolar protein-sorting-associated protein 11) gene. *Vps11* plays a role in vacuole biogenesis during embryogenesis [53]. Dexi7B01G0005290 is a homolog of *AtCLPB1* (HSP101). *AtCLPB1* plays a role in releasing ribosomal RNAs from stress granules for heat stress recovery and plays a role during seed germination (M. [34, 48]. Dexi7B01G0005300 shares similarities with the F-box protein family which regulates diverse cellular processes, such as cell cycle transition, signal transduction, and transcriptional regulation [37]. Besides, we found some genes in other chromosomes with a putative involvement in embryonic development, such as At*XTH28* (homologous to Dexi2B01G0015860), At*KCR1* (resembling to Dexi4A01G0012020), At*ABC17* (akin to Dexi5A01G0007910), At*PDIL1-1* (similar to Dexi5B01G0013640), At*NAGLU* (homologous to Dexi7A01G0021150), and At*MYB98* (sharing similarity with Dexi9B01G0004890). Another set of genes is potentially involved in plant–microbe interaction. For instance, *WAK2* (homologous to Dexi7A01G0021190) is a wall-associated receptor kinase-2 involved in pathogen response and cell expansion during development. A dominant allele of WAK2 activates defense responses, such as the upregulation of numerous genes involved in pathogen resistance and cell wall biogenesis, and ROS accumulation [36]. Another gene involved in ROS regulation is St*RBOHC* (homologous to Dexi8B01G0010430)*.* The related members, At*RBOHD* and At*RBOHF* are key regulators of immunity [46]. At*FMO1* (homologous to Dexi8B01G0010390) is a flavin-dependent monooxygenase, which enhances bacterial and fungal resistance [41, 35]. We did not perform gene ontology (GO) analysis for the biological function as many candidate genes encode for proteins with unknown functions (Table S11). Altogether, we found fonio SNPs to be associated with roles in development, defense response, and cell-wall integrity (Table 1).

## Discussion

Seeds harbor diverse bacterial endophytes, which can be composed of up to 2 billion bacterial cells with a complexity of up to 9000 species [9]. Many studies show that domestication decreased seed microbiome diversity [3, 33]. Fonio, being semi-domesticated, shows high species richness as compared to other crops such as barley, rice, and wheat [4, 14]. A meta-study on 50 plant species worldwide revealed that the seed-core bacterial taxa may include *Pseudomonas*, *Rhizobium*, *Sphingomonas*, *Methylobacterium*, and *Paenibacillus* [51]. We identified these taxa among the core 191 taxa (Table S7) but did not find these taxa (absolute abundance > 1) in all accessions (Table S12). This variation may be attributed to cases where certain bacteria could only be partially assigned (Table S5).

Currently, the role of endophytic bacteria in seeds is linked to plant growth and protection, yet the specific mechanisms remain to be elucidated in detail [30]. We have attempted to elucidate the potential functions of the seed-associated microbiomes in fonio. Our functional analysis revealed the prominence of 35 pathways within distinct groups which included both anabolic and catabolic processes. Notably, we also observed some of these pathways are over/underrepresented by certain groups indicating the endophytes may fine-tune their functions based on the local environment. These results imply that seed-associated endophytes generally support plant growth promotion through nutrient availability and assimilation, with potential adjustments influenced by soil and geo-climatic factors. Therefore, to identify the core endophytic functions across the fonio accessions, we performed the functional profiling of the core microbiome 191 strains, which showed the terms peptidase and inhibitors, secretion systems, and two core component systems. Previously, seed endophytes have been shown to produce biocontrol inhibitors to protect plants from pathogens [39]. Notably, most of the seed endophytic isolates exhibited the production of extracellular enzymes [32] that may assist microorganisms in biofilm formation, combat nematodes, aid in plant nutrient acquisition and in the establishment of microbial communities within plants [21] (Chen et al., 2007) [42]. Also, two core component systems are essential for host-associated bacteria to establish colonization [58]. It is important to note that we have not validated our analysis through alternative methods such as metagenomics or transcriptomics. Together, our analysis is in line with previous studies and suggests the functions of core taxa can be crucial for establishing the endophytic community in the fonio population.

**Fig. 6:**
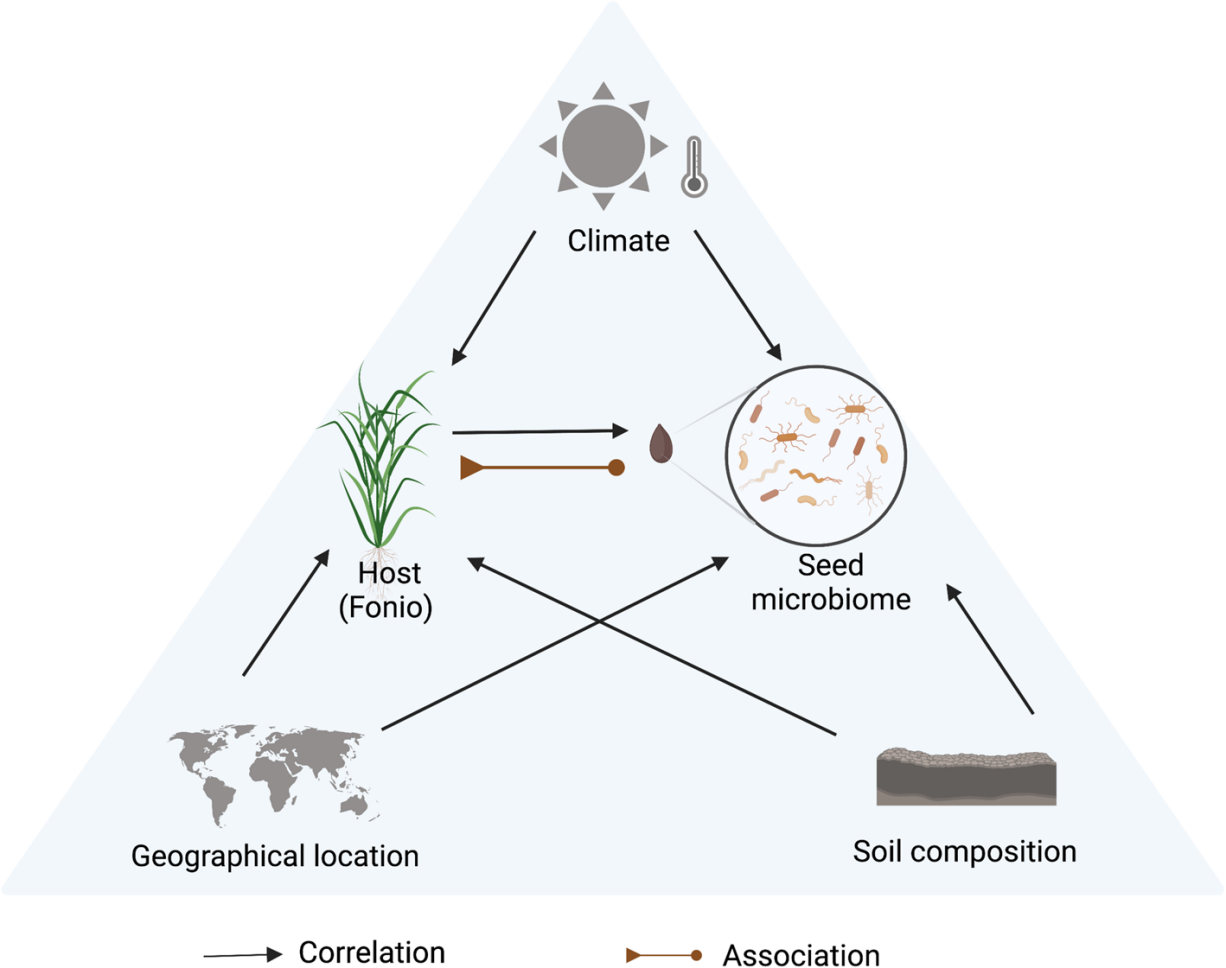
A model proposing climate, geographical location and soil composition may shape host genotype and seed-associated endophytes. A conceptual model illustrating the potential influences of climate, geographical location, soil composition, and the genetic makeup of the host plant to the composition of seed-associated endophytes (*p* > 0.01). Host genetic loci is also associated (*p* = 1e − 5) with seed microbiome diversity

Several studies showed that environmental factors drive rhizosphere and phyllosphere microbiome assembly [16]. Also, host genotypes strongly and weakly shape the leaf and root microbiome, respectively [56]. However, the seed is a bottleneck in the generational continuity of the plant microbiome and may be crucial for plant adaptation. We show in this study that environmental factors and soil properties correlate with the seed microbiome of fonio (Fig. 6), indicating that environmental factors may affect microbial composition and transgenerational transmission.

Previous GWAS showed that host genetic differences influence the composition and diversity of rhizosphere-associated bacteria in Arabidopsis, maize, and Sorghum [19, 40, 43]. Notably, the rhizosphere/leaf/root microbiome is complex and can vary according to environmental conditions [10]. GWAS was also performed with the leaf-associated bacteria of Arabidopsis, maize, and rice [24, 47, 57]. However, to our knowledge, GWAS of seed microbiome traits have not been conducted yet.

The seed-associated endophytic microbes are “selected” in continuity to the next generation; their use may be beneficial. However, seed-to-seed endophyte variability in common beans has also been reported [13]. The variation among the replicates (a pool of six seeds) in seven barley accessions has also been shown (Bziuk et al., 2021b). In our study, among 126 accessions of fonio, 24 accessions showed variation among replicates. Therefore, we used 99 accessions for GWAS analysis and acquired similar peaks with replicates. We found that genetic differences among fonio accessions correlate with the diversity of the seed microbiome. Fonio SNPs associated within and surrounding genes with characterized roles in embryo development, stress/defense response, cell-wall integrity, and nucleic acid metabolism could directly or indirectly affect microbiome composition and diversity. The function of annotated candidate genes highlighted by our study aligns with previous studies. For example, the plant loci responsible for defense affect microbial community variation in the phyllosphere [25] and *RBOHD*, one of the candidate genes in our study, is required for phyllosphere microbiota homeostasis in Arabidopsis [44]. Another GWAS study with the Arabidopsis root microbiome identified host SNPs associated with genes that play roles in immunity, cell wall integrity, and development [11]. One of the genes that shape the bacterial richness and diversity in Arabidopsis is *FMO1* and is also among the candidate genes in our study [12]. We also identified a correlation of microbiome diversity with genes involved in embryonic development, a subject that should receive further attention. Overall, our data suggest that plant genetic loci regulate microbiome composition in a spatially and temporally dependent manner and future experiments with genetic mutants should help to unravel the underlying mechanisms. Taken together, although seed microbiome research is still in its infancy, our work shows that it has great potential in advancing our understanding of the plant-microbiome-environment interaction and in seed microbiome engineering of crops.

### Supplementary Information

**Additional file 1: Fig S1.** Mirobial diversity in 18 fonio accessions (A) Rarefaction plot. (B) Observed features (ASVs). **Fig. S2.** Microbial diversity in 126 fonio accessions across six genetic group and one admixed group (A) Number and distribution of fonio accessions in group (B) Rarefaction plot (C) Histograms of the relative abundances of the top bacterial communities at Phylum (D) Principal components analysis of PICRUSt functional predictions on 126 fonio accessions. Color code is based on the corresponding groups). (E) The flower diagram shows unique and core taxa without admixed group. (F) Functional analysis of the core 191 ASVs for key KEGG pathways. The KO encoded for each subfunction is shown as a bar plot. **Fig. S3. **Phylogenetic tree and absolute abundance of 191 ASVs across the groups represented as stacked bar plot. Each group highlighted with distinct colors. **Fig. S4.** Geographical distribution of fonio accessions showing (A) topsoil sand fraction, (B) topsoil pH, (C) topsoil gravel content, (D) topsoil bulk density, (E) precipitation and (F) temperature. **Fig. S5.** (A) Quantile-Quantile (QQ) plots: the quantile distribution of observed p-values (on the y-axis) versus the quantile distribution of expected*p*-values. (B) The Manhattan plots display the association *p*-value for each SNPs in fonio. The red horizontal lines represent the P= 1e-5 significance threshold. 

**Additional file 2. **

## Data Availability

The raw sequencing data used for bacterial composition are available on NCBI under Submission ID: SUB12628648 and Bioproject ID: PRJNA927337. This study project has been deposited at DDBJ/ENA/GenBank under the accession KHVQ00000000. The version described in this paper is the first version, KHVQ01000000.
