# Supplementary material for: Host genotype, soil composition, and geo-climatic factors shape the fonio seed microbiome
Source: Microbiome. 2024 Jan 17;12:11. doi: 10.1186/s40168-023-01725-5 (PMC10792890; doi:10.1186/s40168-023-01725-5)
Supplement: Supplementary file 2 — Additional file 1: Fig S1. Mirobial diversity in 18 fonio accessions (A) Rarefaction plot. (B) Observed features (ASVs). Fig. S2. Microbial diversity in 126 fonio accessions across six genetic group and one admixed group (A) Number and distribution of fonio accessions in group (B) Rarefaction plot (C) Histograms of the relative abundances of the top bacterial communities at Phylum (D) Principal components analysis of PICRUSt functional predictions on 126 fonio accessions. Color code is based on the corresponding groups). (E) The flower diagram shows unique and core taxa without admixed group. (F) Functional analysis of the core 191 ASVs for key KEGG pathways. The KO encoded for each subfunction is shown as a bar plot. Fig. S3. Phylogenetic tree and absolute abundance of 191 ASVs across the groups represented as stacked bar plot. Each group highlighted with distinct colors. Fig. S4. Geographical distribution of fonio accessions showing (A) topsoil sand fraction, (B) topsoil pH, (C) topsoil gravel content, (D) topsoil bulk density, (E) precipitation and (F) temperature. Fig. S5. (A) Quantile-Quantile (QQ) plots: the quantile distribution of observed p-values (on the y-axis) versus the quantile distribution of expectedp-values. (B) The Manhattan plots display the association p-value for each SNPs in fonio. The red horizontal lines represent the P= 1e-5 significance threshold. [file 40168_2023_1725_MOESM1_ESM.docx]

**Supplementary figures**


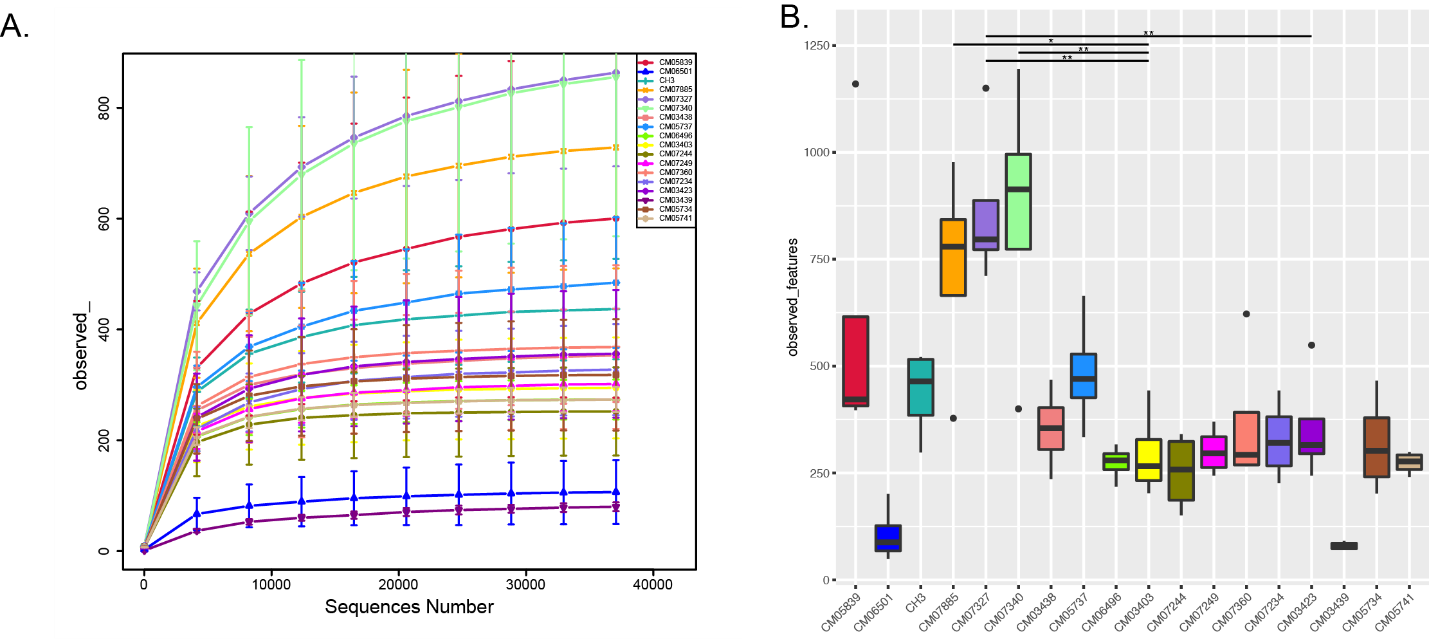


**Fig S1.** Mirobial diversity in 18 fonio accessions (A) Rarefaction plot. (B) Observed features (ASVs)


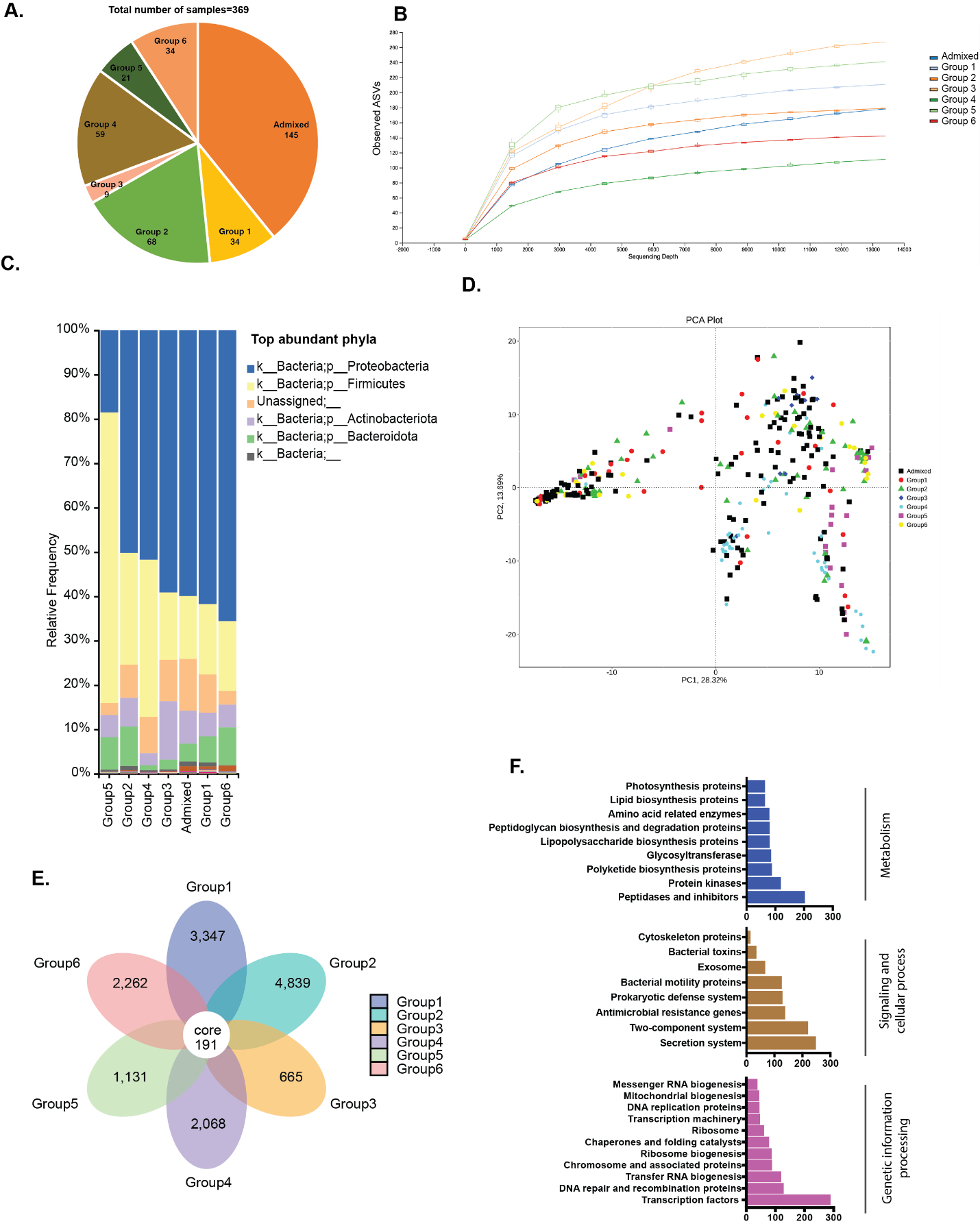


**Fig S2.** Microbial diversity in 126 fonio accessions across six genetic group and one admixed group (A) Number and distribution of fonio accessions in group (B) Rarefaction plot (C) Histograms of the relative abundances of the top bacterial communities at Phylum (D) Principal components analysis of PICRUSt functional predictions on 126 fonio accessions. Color code is based on the corresponding groups). (E) The flower diagram shows unique and core taxa without admixed group. (F) Functional analysis of the core 191 ASVs for key KEGG pathways. The KO encoded for each subfunction is shown as a bar plot.


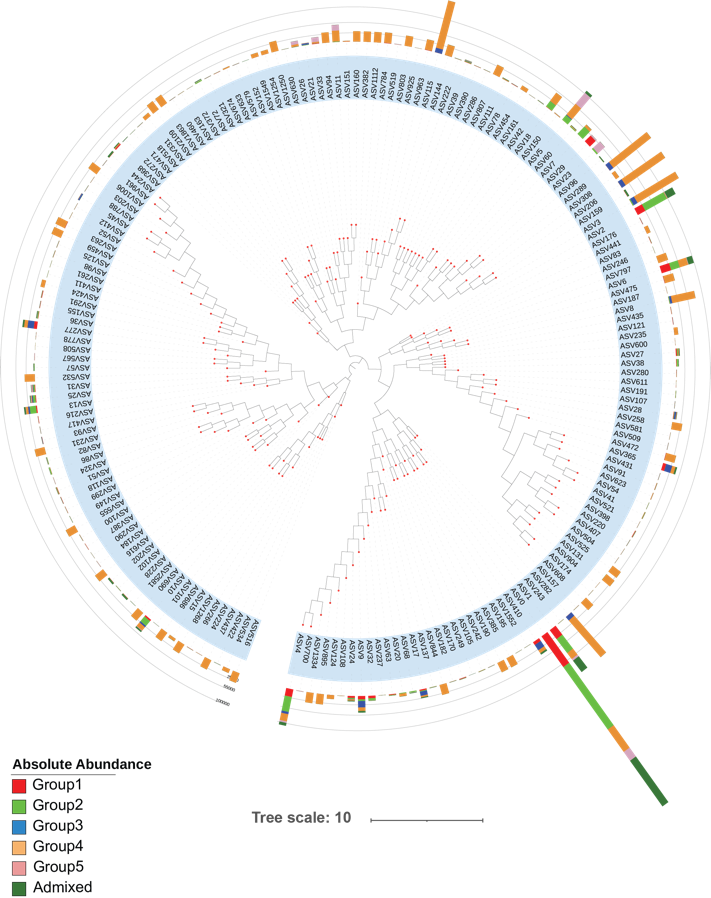


**Fig S3**: Phylogenetic tree and absolute abundance of 191 ASVs across the groups represented as stacked bar plot. Each group highlighted with distinct colors.

_
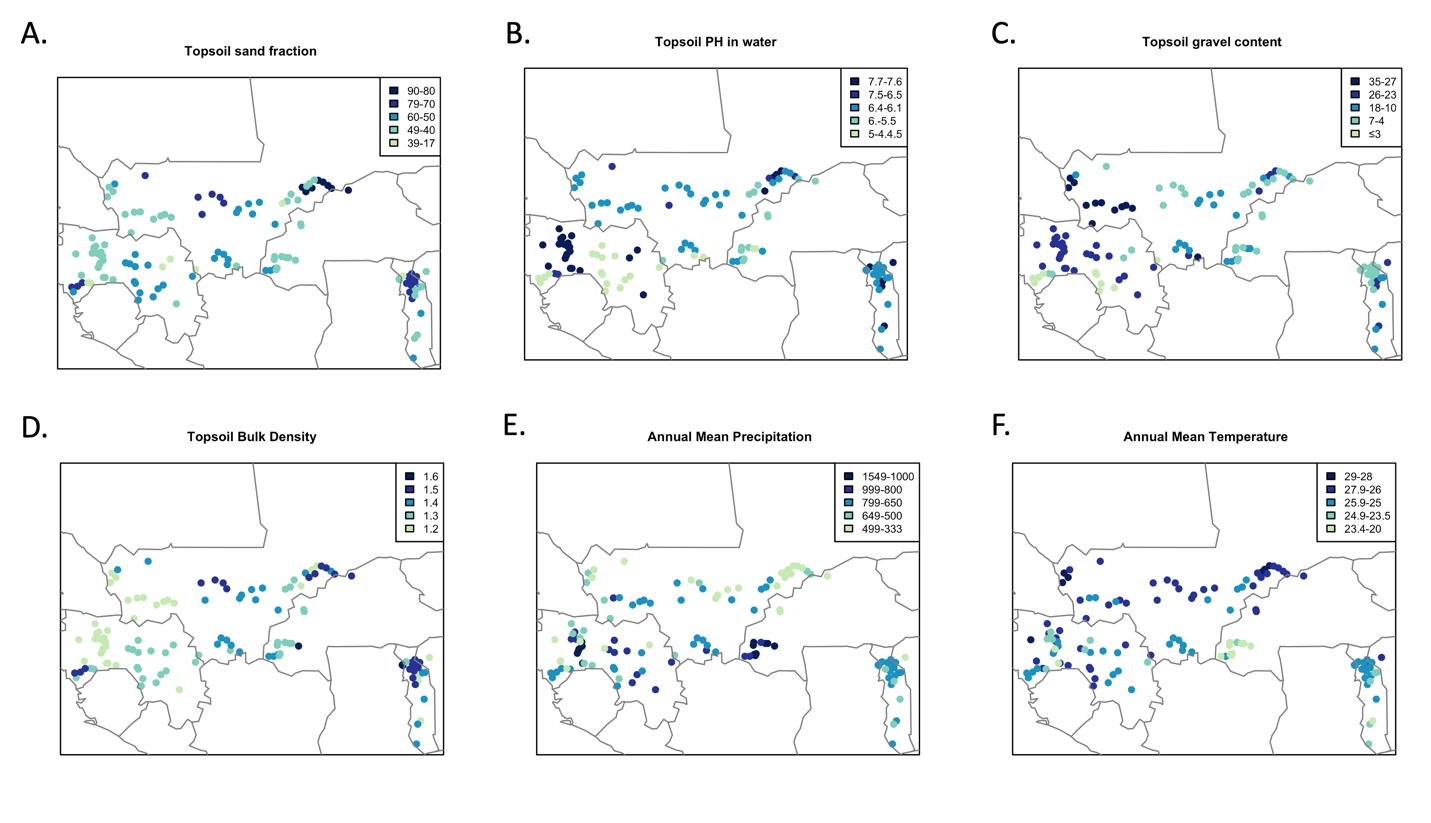
_

**Fig S4.** Geographical distribution of fonio accessions showing (A) topsoil sand fraction, (B) topsoil pH, (C) topsoil gravel content, (D) topsoil bulk density, (E) precipitation and (F) temperature.


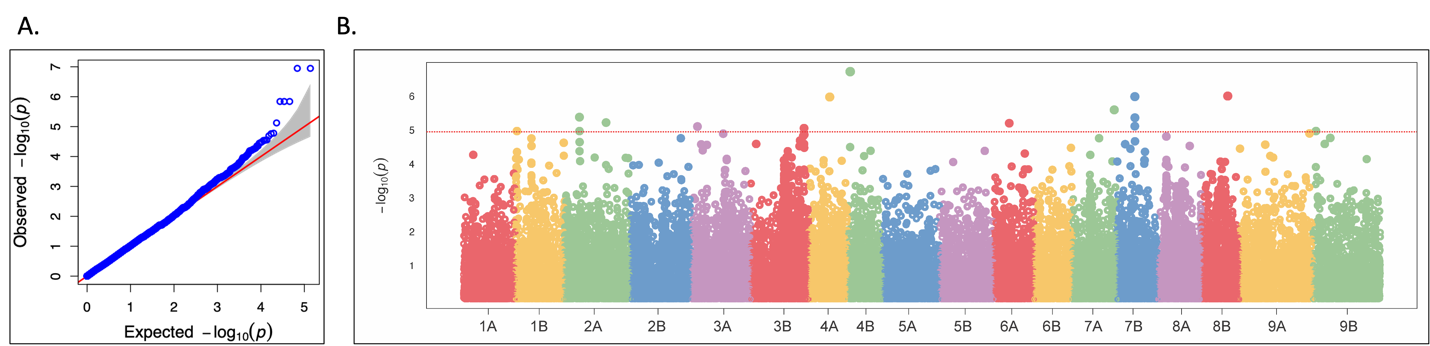


**Fig S5**. (A) Quantile-Quantile (QQ) plots: the quantile distribution of observed p-values (on the y-axis) versus the quantile distribution of expected p-values. (B) The Manhattan plots display the association p-value for each SNPs in fonio. The red horizontal lines represent the P= 1e-5 significance threshold.
